# Visualization of Polyhydroxyalkanoate Accumulated in Waste Activated Sludge

**DOI:** 10.1021/acs.est.3c02381

**Published:** 2023-07-20

**Authors:** Ruizhe Pei, Gerard Vicente-Venegas, Agnieszka Tomaszewska-Porada, Mark C. M. Van Loosdrecht, Robbert Kleerebezem, Alan Werker

**Affiliations:** † Department of Biotechnology, Delft University of Technology, Van der Maasweg 9, 2629 HZ Delft, The Netherlands; ‡ Wetsus, European Centre of Excellence for Sustainable Water Technology, Oostergoweg 9, 8911 MA Leeuwarden, The Netherlands

**Keywords:** polyhydroxyalkanoate (PHA), bioplastic, activated sludge, staining, visualization

## Abstract

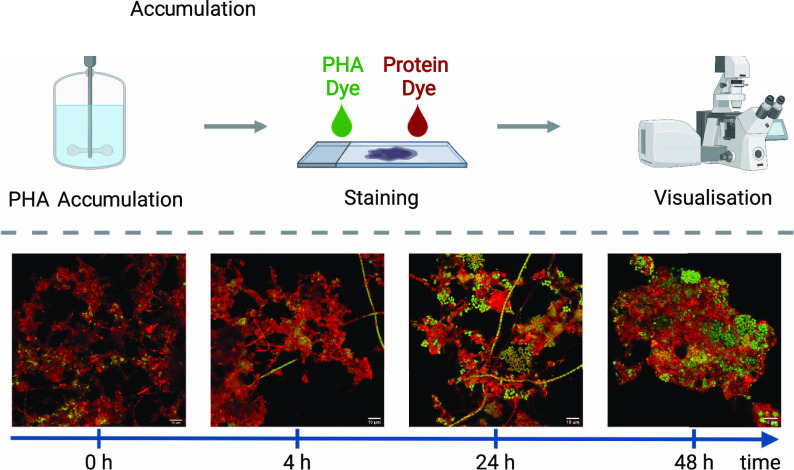

Polyhydroxyalkanoates (PHAs) can be produced with municipal waste activated sludge from biological wastewater treatment processes. Methods of selective fluorescent staining with confocal laser scanning microscopy (CLSM) were developed and optimized to evaluate the distribution of PHA storage activity in this mixed culture activated sludge microbial communities. Selective staining methods were applied to a municipal activated sludge during pilot scale PHA accumulation in replicate experiments. Visualization of stained flocs revealed that a significant but limited fraction of the biomass was engaged with PHA accumulation. Accumulated PHA granules were furthermore heterogeneously distributed within and between flocs. These observations suggested that the PHA content for the bacteria storing PHAs was significantly higher than the average PHA content measured for the biomass as a whole. Optimized staining methods provided high acuity for imaging of PHA distribution when compared to other methods reported in the literature. Selective staining methods were sufficient to resolve and distinguish between distinctly different morphotypes in the biomass, and these observations of distinctions have interpreted implications for PHA recovery methods. Visualization tools facilitate meaningful insights for advancements of activated sludge processes where systematic observations, as applied in the present work, can reveal underlying details of structure–function relationships.

## Abstract 2

Municipal activated sludge can accumulate PHA, but different biomass PHA content limits are often found. In this work, staining methods to visualize PHA granule distribution and specific biomass components (DNA, RNA, and protein) were developed, optimized, and applied for a case study.

## 1 Introduction

Polyhydroxyalkanoates (PHAs) are a family of polyesters produced by a broad range of microorganisms. They are stored as intracellular granules and provide intermediate reservoirs for the supply of carbon and energy.^1−3^ Microorganisms that accumulate PHA do so typically when environmental conditions result in stresses, such as a lack of an essential nutrient, while organic carbon is available in excess.^4−6^ PHAs recovered from PHA-rich biomass exhibit thermoplastic and mechanical properties similar to petroleum-based polyesters commonly used today.^7,8^ Distinct from petroleum-derived polymers, PHAs are bio-based and biodegradable. Research and development efforts advance to realize the potential for PHA production using wastewater as feedstock and microbial community-based production methods.^9,10^

Microbial community-based PHA production methods may be categorized as enrichment accumulation or direct accumulation, depending on the source of the biomass.^10^ Enrichment accumulation starts with specifically cultivating a biomass. A feast–famine selection pressure is typically applied to produce a bacterial biomass using volatile fatty acid (VFA)-rich feedstocks to enrich for PHA storing bacteria.^11−13^ The surplus enriched functional biomass is then fed with VFA-rich substrates in a separate PHA accumulation bioprocess to reach its maximum PHA content.

Alternatively, direct accumulation exploits activated sludge from municipal and industrial biological wastewater treatment plants (WWTPs). In a PHA accumulation process, the waste activated sludge, without further enrichment, is directly fed with a VFA-rich feedstock to reach its maximum PHA content.^14^ Wastewater activated sludge has exhibited significant PHA-accumulating potential.^15−20^ This significant level of PHA-accumulating potential in activated sludge can rise because the environmental conditions in the WWTP are inherently or purposefully engineered to be selective to favor survival for PHA storing phenotypes.^3^ The selectivity can be due to a feast–famine effect caused by the plug flow of the wastewater treatment lanes or day and night regime for the influent wastewater loading.

The PHA content in a dried biomass sample is readily quantified. Biomass PHA content is frequently reported as a part of accumulation performance assessments.^9,10^ Enrichment accumulation methods at the laboratory scale have achieved a biomass PHA content of 90% gPHA/gVSS.^13^ However, biomass PHA content under industrial conditions is often lower and can range between 40 and 80% gPHA/gVSS.^10,21,22^ In these enrichment cultures, most of the biomass typically stores PHA. Thus, enrichment culture biomass is expected to have a high degree of enrichment for PHA-accumulating microorganisms. As an example, enrichment accumulation was applied using the fermented organic fraction of municipal solid waste. A biomass PHA content of 46 ± 5% gPHA/gVSS was achieved, and staining revealed a very high degree of enrichment.^23,24^ Therefore, the PHA accumulation potential, or maximum biomass PHA content reached for enrichment cultures with a high degree of enrichment (∼100%), estimates what the individual species of bacteria in the biomass can achieve on average.

With direct accumulation, surplus activated sludge has also resulted in biomass PHA content of about 50% gPHA/gVSS.^25,26^ However, in these cases and in general in the research literature, biomass PHA contents are not reported together with information of the degree of enrichment. Determination of biomass PHA content alone is insufficient to evaluate both the inherent degree of enrichment for PHA-accumulating microbes and average performance for individual cell accumulation potential. Lower or higher biomass PHA content for activated sludge can be due to lower or higher degree of enrichment or selected PHA-accumulating bacteria with a lower or higher maximal PHA content. Insight requires information on the distribution of the PHA storing phenotype. This distribution defines the property of the degree of enrichment of activated sludge. To explore the property of degree of enrichment, visualization methods were necessary, and this became a focus of development within the larger research goals of establishing optimal generic production methods of PHAs by direct accumulation with surplus municipal activated sludge.

Staining of PHAs with counterstaining using different dyes can selectively visualize the intracellular PHA granules and their distributions in the biomass.^5,27^ The most commonly applied dyes are Nile red, Nile blue A, and Sudan black B.^5,27^ Bright-field microscopy or a counterstain for DNA via DAPI have also been applied in order to assess for the extent and distribution of polymer storage activity in the biomass. PHA granule staining can be complemented furthermore with fluorescence *in situ* hybridization (FISH) targeting 16S rRNA. FISH can reveal the consequent extent of microbial activity via a general bacterial probe EUB338 mix or can identify the abundance of specific microorganisms via specifically designed probes.^28,29^

In the present study, complementary counterstaining methods were optimized specifically for municipal activated sludge biomass from a full-scale WWTP. The methods were developed using samples taken during 48 h fed-batch PHA direct accumulation experiments at a pilot scale. Different promising staining and confocal laser scanning microscopy (CLSM) strategies were initially screened. The best approaches were then optimized into standardized protocols. Staining methods included Nile blue A, Nile red, BODIPY 493/503 (BODIPY), DAPI, bright field, protein staining via SYPRO Red, and FISH. Ultimately, optimal outcomes were developed by combining BODIPY and SYPRO Red, or BODIPY, FISH, and DAPI. Systematic evaluations were then made in the first application of the protocols that were developed, and these are reported herein.

## 2 Materials and Methods

### 2.1 PHA Accumulation and Sample Fixation

PHA accumulation experiments were performed in a jacketed stainless steel 200 L pilot scale reactor with 167 L working volume. Influent volume flowed out as clarified effluent via a 16 L gravity settler, and flow was actively recirculated between the main vessel and the settler. Temperature was maintained at 25 °C, and mechanical mixing was constant at 230 rpm with a standard impeller. Aeration was via a membrane disk and constant at 50 L/min.

Waste activated sludge was from Bath WWTP (Rilland-Bath, the Netherlands), which treats 470,000 person equivalents. The WWTP handles a mixture of municipal and industrial influent wastewater with screening and primary treatment. Secondary treatment is by tanks in series creating plug flow in a modified Ludzack–Ettinger activated sludge biological process (10 independent parallel treatment lanes with 20 days solids retention time). Phosphorus removal is by precipitation using FeCl_3_. Previous studies have demonstrated consistent performance in PHA production by direct accumulation.^14^ Fresh grab samples of gravity belt thickened waste activated sludge (56.7 gTS/L) were delivered biweekly by courier on the same day. This source biomass was stored at 5 °C for no more than 2 weeks, pending its use in accumulation experiments.

Prior to each accumulation experiment, the pilot reactor was first loaded with a determined weight of the thickened waste activated sludge that was then brought to a starting mixed liquor suspended solids concentration of about 2.5 gVSS/L by dilution with tap water. The reactor was brought up to 25 °C with constant mixing and aeration. Experiments were started after about 12 h once a steady state level of endogenous respiration was reached.

The feedstock was 20 gCOD/L acetic acid (VWR, the Netherlands) with added NH_4_Cl and KH_2_PO_4_ (VWR, the Netherlands) for a COD:N:P (by weight) of 100:1:0.05. The pH was adjusted to 5.0 ± 0.5 with NaOH (VWR, the Netherlands). Influent was given in discrete pulses of a fixed volume. The pulse volume targeted a peak substrate concentration of 100 mgCOD/L for each input.

Pulse input feedback control was by respiration level monitoring based on dissolved oxygen (DO) measurements (JUMO ecoLine O-DO, JUMO GmbH & Co. KG, Germany) as previously described by Werker et al.^9^ The process started with an acclimation step.^30^ Acclimation comprised three feast–famine cycles. This meant that the first three pulse inputs were provided, wherein DO was monitored to measure the (feast) time for the added substrate consumption. A famine time of 3 times the estimated feast time was then imposed before the next pulse input cycle. After acclimation, the accumulation process was automatically started. During accumulation, feed pulses were given without any delay from one pulse to the next. Thus, the accumulation process was characterized by a feed-on-demand feast with a series of input pulses over 48 h. In total, three replicate PHA accumulations, referred to as ACC 1, ACC 2, and ACC 3, were operated using three different batches of activated sludge.

Over the course of each accumulation experiment, duplicate mixed liquor grab samples (50 mL) were taken at selected time points. One of the grab samples was acidified with addition of 98% H_2_SO_4_ (VWR, the Netherlands) to pH 2 and then centrifuged at 3248 RCF for 5 min at 4 °C (Beckman Coulter, CA). The suspended solids pellet was separated from the supernatant and dried overnight at 105 °C. Dried pellets were ground and assessed by thermal gravimetric analysis (TGA 2, Metller Toledo, Switzerland) for biomass PHA content.^31^ The duplicate grab samples were fixed directly with formaldehyde.^28,29^ 5 mL subsamples were combined with 5 mL of 1× phosphate-buffered saline (PBS) (PanReac AppliChem, ITW Reagents, Spain) and 1.1 mL of 37% formaldehyde (Sigma-Aldrich, the Netherlands), resulting in the 3.7% final formaldehyde concentration. After incubation (∼3 h or 12 h) at 4 °C, four rinse cycles were performed with 15 mL of 1× PBS by centrifugation (3248 RCF for 5 min at 4 °C), decantation, and resuspension. The pellet was then resuspended with 10 mL of 1× PBS and 10 mL of pure ethanol (VWR, the Netherlands) and stored at 5 °C. After first analyses, fixed samples were preserved by storage at −20 °C for later use.

### 2.2 Staining Methods and Microscopy

#### 2.2.1 PHA Staining and Biomass Counterstaining

Nile blue A staining was with 10 μL of fixed sample dispensed onto a glass slide, followed by drying at 50 °C on a heating plate.^32^ The dried slide was dipped into a 1% (v/v) solution of aqueous Nile blue A and then incubated at 55 °C for 10 min. The slide was rinsed first with Milli-Q water (Merck, Germany) and then with 8% acetic acid (VWR, the Netherlands) for 1 min. The slide with the stained sample was finally dried, then mounted with VECTASHIELD HardSet Antifade Mounting Medium H-1400-10 (Vectashield) (Vector Laboratories, CA), and sealed.

For Nile red staining, 1 mL of a fixed activated sludge sample was centrifuged at 12,000 RCF for 5 min. The supernatant was discarded, and the pellet was resuspended with 1 mL of Milli-Q water. Nile red solution was added to reach selected concentrations ranging between 1.6 and 7.6 μg/mL. The sample was incubated at room temperature for 30 min. The incubated sample was centrifuged (12,000 RCF 5 min), and the harvested pellet was resuspended with 1 mL of Milli-Q water and mixed thoroughly. 10 μL of the stained and well-mixed sample was deposited to a clean glass slide, dried, mounted with Vectashield, and sealed.

BODIPY 493/503 (BODIPY) (Thermo Fisher Scientific, MA) for PHA granule staining was combined with protein staining via SYPRO Red originally 5000× concentrated (Thermo Fisher Scientific, MA). Glass microscope slides with ten 5 mm diameter reaction wells were used (Paul Marienfeld GmbH & Co.KG, Germany). In each well, 5 μL of the fixed sample was dispensed, followed by 0.5 μL of BODIPY with a concentration of 2 ng/μL. Then, 0.5 μL of 100 times diluted SYPRO Red was added. The slide was then completely dried at 46 °C for at least 1 h. The prepared slide was finally washed with Milli-Q water, dried with compressed air, mounted with Vectashield, and sealed. A negative control well was also included for each slide with the sample but with no dye applied.

#### 2.2.2 Fluorescence *In Situ* Hybridization Combined with PHA Staining

Fluorescence *In Situ* Hybridization (FISH) was applied using EUB338-I with the sequence 5′ GCT GCC TCC CGT AGG AGT 3′ labeled with Cy5 fluorophore (biomers.net GmbH, Germany). In a parallel study, the microbial community before and after the PHA accumulation using activated sludge from Bath WWTP was profiled by sequencing of the 16S rRNA gene.^33^ Using these sequencing results as the database, the coverage of EUB338-I probe was tested in silico by ARB software.^34^ EUB338-I probe alone showed high coverage; therefore, EUB338-II and EUB338-III from EUBmix were not applied in the current study. FISH was combined with PHA staining via BODIPY and DNA staining via DAPI.^28,29^ Glass microscope slides with ten 5 mm diameter reaction wells were used again. 5 μL of fixed samples were loaded to each well, heat-fixed, and then dehydrated with 50, 80, and 100% ethanol. Then, 10 μL of hybridization buffer with 35% formamide was added, followed by 0.5 μL of EUB338-I, BODIPY, and DAPI with a concentration of 50, 2, and 250 ng/μL, respectively.

The slide was incubated in a hybridization chamber at 46 °C for 1.5 h. The sides with hybridized sample were then washed and incubated in a prewarmed buffer solution at 48 °C for 15 min. After subsequent washing with cold Milli-Q water, the slide was dried, mounted with Vectashield, and sealed. A negative control well was also included for each slide with the sample but with no dye applied.

#### 2.2.3 Confocal Laser Scanning and Epifluorescence Microscopy

Bright-field observations were made by a light microscope BX43 equipped with a DP80 camera (Olympus, Japan) and by a confocal laser scanning microscope LSM 880 (Carl Zeiss, Germany). For the evaluation of selectively stained biomass, the LSM 880 with a Plan-Apochromat 63×/1.4 Oil DIC M27 objective (Carl Zeiss, Germany) was used. The stained sample areas were always surveyed first. Then, 11 fields of view with areas containing floc structures were selected randomly for a sequence of images. For each field of view, respective fluorescence dye emission signals were visualized at the optimum excitation wavelengths. Images from each excitation wavelength were recorded into separate image channels with 16 scans averaged at a 16-bit depth.

Excitation wavelengths were: diode 405-30 laser at 405 nm for DAPI, argon laser at 488 nm for BODIPY, DPSS 561-10 laser at 561 nm for SYPRO Red, and HeNe633 laser at 633 nm for Cy5. Imaging conditions, laser power, and gain were constant for the set of images from each reaction well. Composite images were generated by overlaying channels from the same field of view.

Similar laser power and gain levels were also used among the set of reaction wells for a given slide. The negative control wells were evaluated employing similar imaging parameters to assess any potential artifacts that could come due to autofluorescence. Images were analyzed using Fiji ImageJ (ImageJ2, ver 1.52p).

## 3 Results and Discussion

The method development for the present investigation started with evaluation of staining methods for PHA granules accumulated in waste activated sludge (Nile blue A, Nile red, and BODIPY). Based on the resolution of PHA granules and the excitation–emission specificity of the dyes, BODIPY was selected. Then, the different incubation conditions and dye concentrations were evaluated to identify the optimal staining conditions for PHA visualization. After selection and tuning of BODIPY as the preferred stain, complementary counterstaining methods for other biomass components were established to visualize PHA distribution with respect to biomass as indicated by protein, DNA, and RNA. A standard protocol was implemented and then applied to observe the development of PHA granule distribution in waste activated sludge based on replicated direct accumulation experiments. Qualitative observations were made for the distribution of PHA accumulation activity in the biomass with respect to the general distribution of biomass activity. Morphology of PHA storing communities, individual cells, and granules within cells were assessed.

### 3.1 Selection of PHA Staining and Counterstaining Methods

For the visualization of PHA granule distribution in the biomass, Nile blue A, Nile red, and Sudan black B have been widely applied.^5,27^ In this study, preliminary work was conducted with Nile blue A and Nile red using selected samples of PHA-rich biomass collected before and after 24 h of accumulation. No significant PHA staining signal was detected in the fresh waste activated sludge sample. After 24 h of accumulation, Nile blue A stained for PHA (Figure S1). However, the signal from the stained PHA granules was found to be diffuse. In this way, the image acuity was deemed to be insufficient in its definition and detail for the intended evaluations of the PHA distribution in the biomass. Additionally, it was found that fluorescence signal was emitted in a very broad laser excitation range from 405 nm to more than 600 nm. This wide excitation breadth meant that complementary staining could not be readily applied without a risk for significant cross interference if multiple of biomass components were to be independently revealed.

Nile red gave a better quality in the resolution for staining PHA granules compared to Nile blue A (Figure S1). This better quality fit with expectations based on the literature.^35^ However, Nile red similarly exhibited a broad wavelength range for the dye excitation. Therefore, neither of the Nile dyes was found to be suitable for a strategy involving counterstaining and imaging for selected biomass components in isolation from one and the other. An alternative PHA granule staining method with similar or better acuity but with narrower excitation–emission characteristics was required.

Developments were directed to evaluate BODIPY 493/503, a lipophilic dye, which has also been applied to selectively stain PHA granules.^5^ An optimal BODIPY PHA staining procedure for the PHA-rich municipal activated sludge was developed. Variations of sample incubation temperature, incubation time, and applied BODIPY concentration were tested and compared based on the influence of the protocols on the fluorescence signal quality. Incubation trials were carried out at room temperature, 40 and 46 °C and with incubation times from 10 min up until all of the liquid evaporated after about 1 h. It was found that maintaining incubation at 46 °C until all of the liquid evaporated yielded the best results in terms of signal quality and stability.

BODIPY concentrations in the range of 0.2–20 ng/μL were evaluated. When the BODIPY concentrations were lower, the fluorescence signal was weak and bleached out during laser excitation within a few seconds. Rapid signal bleaching prevented capturing images with a better resolution by averaging multiple scans, making Z stacking composite images to reveal three-dimensional (3D) structures, and reusing the slides for repeated analyses. When the applied BODIPY concentration was too high (>2 ng/μL), the image signal remained stable even for prolonged laser excitation. However, image quality in acuity suffered due to overexposure. This meant that finer details of the PHA granule distribution were not distinguishable. Excessive applied BODIPY loading also left unwanted dye residue after the washing steps. This residue generated a diffuse background signal, which caused a loss in image definition. The balance for good fluorescence stability and quality to resolve details was found at 2 ng/μL.

Excitation wavelengths of 405, 488, 561, and 633 nm were then examined to identify the optimal excitation wavelength. A strong fluorescence signal was obtained just at 488 nm. Thus, the other wavelengths could be used for other kinds of stains to independently reveal other biomass features. A narrow excitation and emission spectrum range was essential to enable PHA granule staining in combination with the selective fluorescent staining of other biomass components.

**Figure 1:**
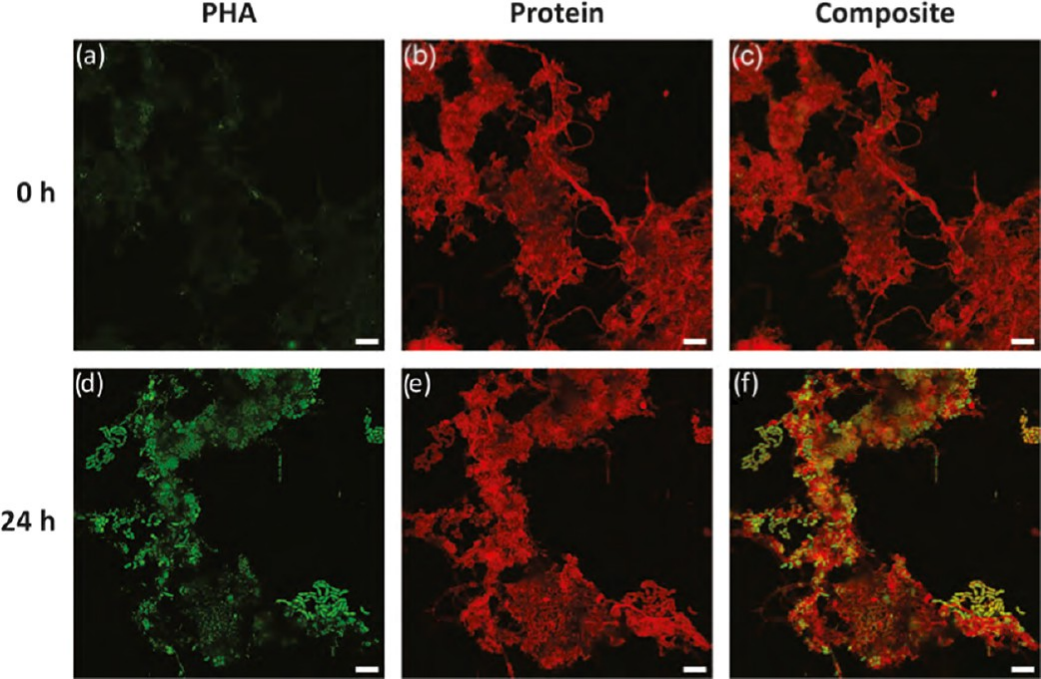
Visualization of PHA accumulation in activated sludge by staining PHA with BODIPY (green) and protein SYPRO Red (red). Samples at the start of accumulation (a–c) and after 24 h of accumulation (d–f). Scale bars represent 10 μm.

BODIPY is a lipophilic dye similar to Nile blue A and Nile red. This characteristic suggests that BODIPY cannot be assumed to be uniquely specific to PHA granules. Other lipophilic features of the biomass, the cell chemical structure, or other storage compounds, such as intracellular lipid droplets, could be coincidentally stained.^36^ Thus, for each case, the specificity of a dye needs to be examined and cross-validated with complementary measurement methods. The specificity of BODIPY toward PHA granules in the municipal waste activated sludge was examined in comparison with PHA quantification using TGA. The biomass samples before and after the PHA accumulation experiments were stained with BODIPY (Figure 1). The same staining protocol was applied, and images were acquired using similar imaging parameters. Minor levels of point source fluorescence were observed in the biomass before accumulation (Figure 1a–c). This initial signal was similar to the experience with Nile blue A and Nile red. The observation fits with expectations due to initial minor levels of measured PHA content of around 0.01 gPHA/gVSS in the waste activated sludge. In contrast, the PHA-specific signal after 24 h of PHA accumulation was intense and widely distributed throughout the biomass flocs (Figure 1d–f). PHA accumulation was carried out using acetate as the sole substrate, and the applied process method favors PHA accumulation. Other intracellular storage compounds, such as lipid droplets, were not expected or otherwise reported. The volatile suspended solids and PHA content was followed over time during the accumulation. The net differences between volatile suspended solids and PHA mass represented the non-PHA organic fractions, including cells, extracellular polymeric substances (EPS), and other intracellular storage compounds such as lipid droplets. In these three replicate accumulation experiments, the non-PHA organic fractions remained constant, suggesting no measurable active growth or accumulation of other storage compounds. On the other hand, BODIPY was observed to follow the development of a staining signal corresponding directly to significant PHA accumulation. There were no signs of the stain illuminating other biomass generic or specific features such as cell membrane phospholipid structures or floc EPS (Figure 1a–c). Therefore, BODIPY was considered specific toward PHA in this study.

Revealing distribution of PHA granules within the biomass morphological structures required a counterstain. The counterstain should show the complement of the non-PHA biomass. Bright field or staining for specific cellular compounds could serve this goal. The latter was preferred due to an anticipated diversity in floc morphology and suspended solids chemical character for municipal activated sludge. Therefore, methods were developed to evaluate accumulated PHA distribution with reference to specific, strategic, and stainable microbial cell chemical targets. Biomass protein and DNA were the selected targets to represent non-PHA biomass because of their ubiquitous presence, with contents of typically 55 and 3.1 wt %, respectively.^37^

SYPRO Red was evaluated for generic cellular protein staining with dilutions from 1 to 5000 times of the original reagent (manufacture given as 5000× concentrated). An optimum balance of fluorescence stability and signal resolution was found at a 100 times dye dilution. Similarly, DAPI for DNA staining was found to be optimal at 250 ng/μL. Non-PHA biomass staining was compared to floc structures as resolved using bright-field microscopy. Figure S2 illustrates how DNA and protein component staining overlap consistently with the bright-field floc images. The consistently overlapping areas supported that DNA and protein staining could effectively provide a complimentary fluorescent signal from which to evaluate PHA distributions visualized by BODIPY. CLSM images from DNA and protein staining Red offered a degree of resolution and definition for individual cell morphology that was not accessible with the bright-field images. This improved resolution of details allowed for confidence in making morphological evaluations of the PHA storing organisms in the biomass.

Negative control wells for each slide with samples prepared without any added dye were evaluated using similarly applied parameters for image acquisition and post analysis. Negligible autofluorescence was observed with excitation wavelengths for BODIPY (PHA) and SYPRO Red (protein). Thus, signal evaluations, including pixel area counts, were not biased by background signal noise. However, DAPI (DNA) stain excitation at 405 nm did result in a low level of autofluorescence for the unstained biomass. DNA staining by DAPI was to be indicative of overall non-PHA biomass areas and distributions. A weak redundant autofluorescence under the stronger positive signal from DNA staining of biomass was therefore assumed to not introduce a bias for the purposes of the present investigation. Thus, the staining combinations with optimum conditions for BODIPY (PHA) with SYPRO Red (protein), or BODIPY (PHA) with DAPI (DNA), were applied further toward a routine protocol for staining and PHA distribution image analyses.

### 3.2 Application of Selective Staining in an Activated Sludge: PHA Distribution Development

**Figure 2:**
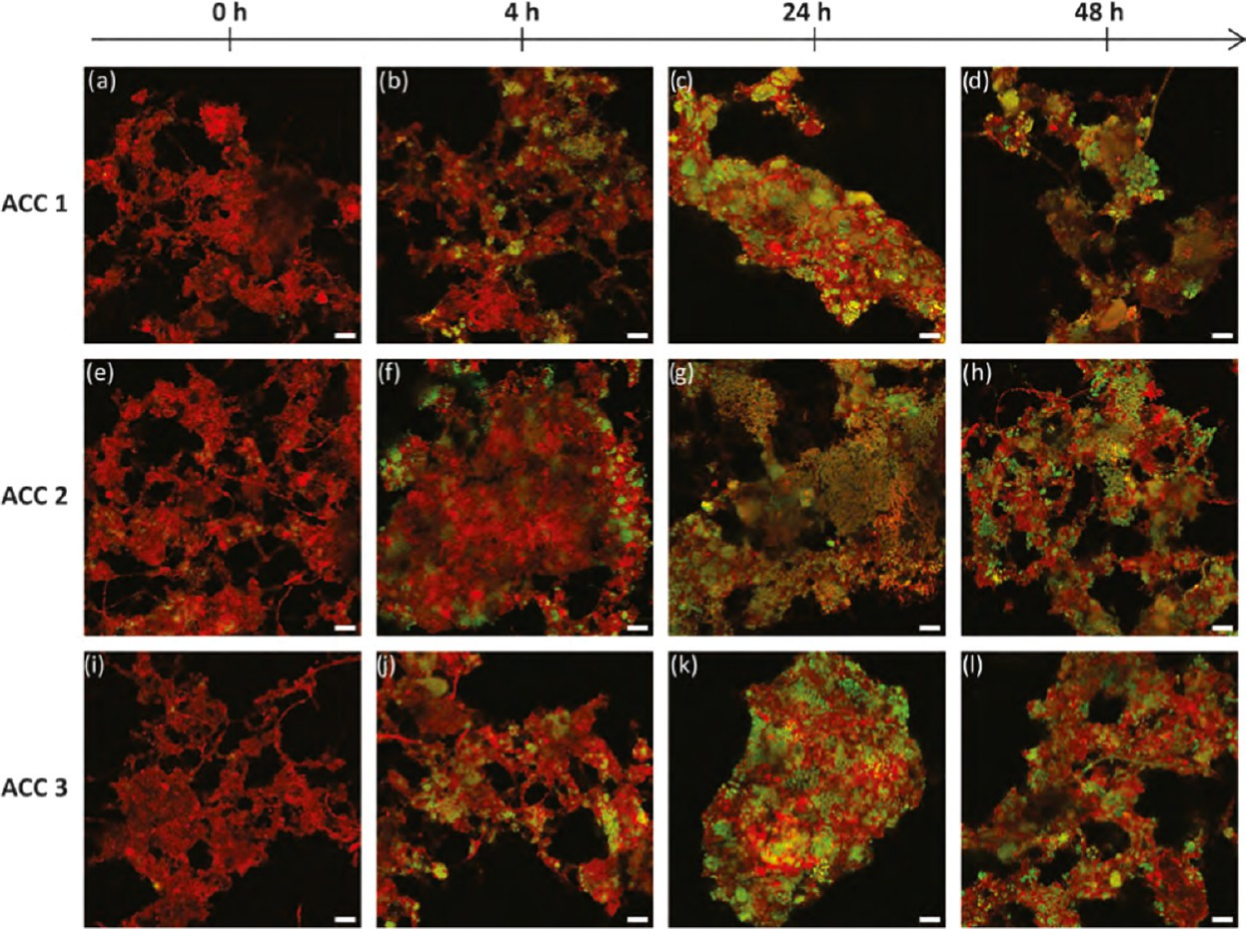
Direct accumulation replicate experiments with developments over 48 h revealed by combined PHA (green) and protein (red) staining. Yellow represents the overlapping signal between PHA and protein staining. Samples from replicate accumulations ACC 1 (a–d), ACC 2 (e–h), and ACC 3 (i–l). Scale bars represent 10 μm.

**Figure 3:**
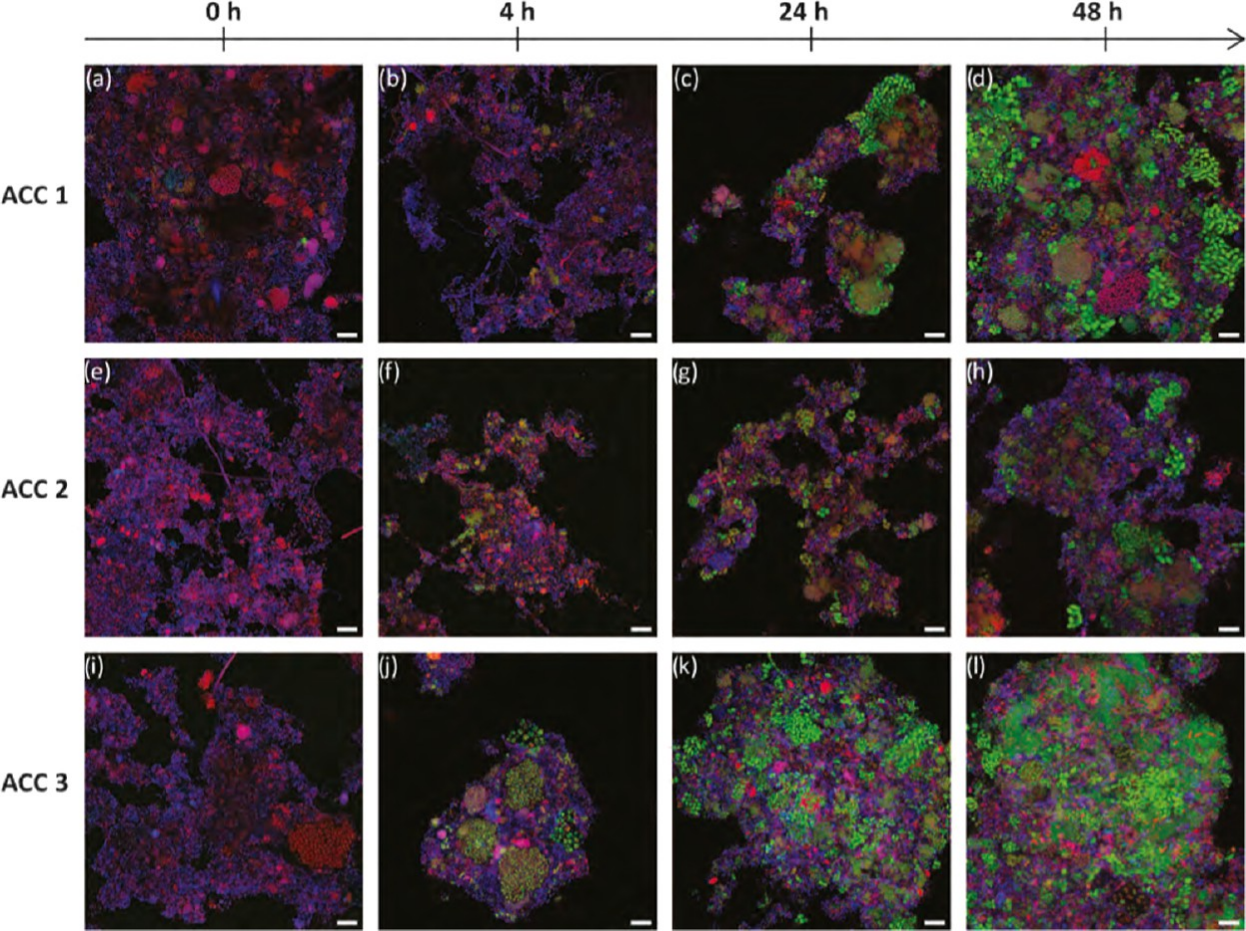
Direct accumulation replicate experiments with developments over 48 h revealed by combined PHA (green), DNA (blue), and FISH (red). Magenta represents the overlapping signal between RNA and DNA staining. Yellow represents the overlapping signal between RNA and PHA staining. Samples from replicate accumulation ACC 1 (a–d), ACC 2 (e–h), and ACC 3 (i–l). Scale bars represent 10 μm.

As a case study, the empirically established optimal selective staining methods were applied to follow an activated sludge during replicated PHA accumulation production process campaigns. During the accumulation process, the biomass PHA content increased with pulse feeding of the acetic acid-rich feedstock. A maximum plateau level of biomass PHA content was approached asymptotically by 27 h. Three replicate accumulation experiments gave similar plateau biomass PHA content levels of 0.48 ± 0.02 gPHA/gVSS. The development of PHA distribution over time was followed also from samples fixed and stained using BODIPY and SYPRO Red for PHA and protein, respectively. The process step of acclimation before accumulation has repeatedly been shown to increase the maximal accumulation potential for a waste activated sludge.^30^ After acclimation, some PHA storage could already be observed (Figures 2a,e,i and 3a,e,i). The initial PHA granule distribution was not homogeneous. Patches of cell clusters with PHA were present after the third feast–famine cycle. Thus, some microorganisms stored PHA due to the applied three acclimation feast periods, while not all of the polymer was metabolized in the time given during subsequent respectively applied acclimation famine periods. In particular, free-living long filamentous bacteria were noted to be the early accumulating organisms within the biomass. This morphological distinction could suggest potential selective winners due to a faster substrate uptake rate.^38^ It could also suggest differential rates of stored polymer metabolism during famine.^39^

The pulse-wise feeding rate was coupled to the substrate uptake rate based on dissolved oxygen concentration as a proxy for the biomass oxygen uptake rate.^9^ The relative area of floc PHA granule coverage with respect to the nonstained biomass increased markedly over the course of the accumulation process. These observations were readily facilitated due to protein counterstaining. For example, Figure 2 shows typical outcomes during accumulation after 0 h and 48 h. However, even by 48 h, not all of the biomass floc area became covered with stored PHA (Figure 2d,h,l). Biomass in municipal activated sludge may have significant fractions of inert solids, non-PHA storing organisms, as well as dormant microorganisms. In dedicated PHA enrichment cultures, virtually all bacterial cells have been observed to accumulate PHA.^22,24^ The degree of enrichment can be up to essentially 100 percent for these highly functionalized enrichment accumulation biomass. Such a high level of enrichment is not expected for municipal waste activated sludge.

PHA and protein biomass staining methods were combined to help visualize cell morphology and the heterogeneous nature of PHA granule distribution during direct accumulation. 16S rRNA-based microbial activity measurements and phylogenetic identification based on specific FISH probes were also considered. The goal was to associate specific genotypes in the activated sludge with PHA production. The development started with FISH using the EUB338-1 probe. However, combining FISH with PHA and protein was challenging due to fluorescent emission signal interference with either BODIPY or SYPRO Red signals excited at 488 nm and at 561 nm, respectively. A fluorophore in conjunction with a FISH probe was sought with an excitation wavelength in the UV range (405 nm). Thus, fluorophores, including Alexa 405, Atto 425, Eterneon 393/523, and Pacific blue conjunct with EUB338-I at both 3′ and 5′ ends, were evaluated. Labeled probes were applied at concentrations in the range from 50 to 500 ng/L. Probe concentrations were tested in combination within ranges of hybridization times (1 h to overnight), hybridization temperatures (37–50 °C), and formamide concentrations (0–50%). Unfortunately, under all of the tested combinations of conditions, low quantum yields were obtained from the dyes excited in the ultraviolet range. The low yield limited the ability to generate sufficient fluorescence signal to distinguish with respect to an autofluorescence negative control using the same imaging parameters. Compared with dyes excited in the ultraviolet range, fluorophores that are excited with higher wavelengths naturally have a higher quantum yield. Therefore, Cy5-labeled FISH probe was tested. A strong and distinct signal was obtained, suggesting that the previous issue with FISH was due to the signal of the fluorophores rather than from the permeabilization of cells or the procedures of FISH. Since it is challenging to combine PHA, RNA, and protein staining, a combination of PHA, RNA, and DNA staining was further evaluated as an alternative approach.

The combination of PHA, RNA, and DNA staining was performed using BODIPY, EUB338-I (Cy5-labeled), and DAPI. FISH with DNA counterstaining was applied to reveal the distribution of cellular viability with activity in the biomass with respect to zones in the distribution of PHA storing activity. Figure 3 illustrates how the PHA (green) and DNA (blue) counterstaining approach gave similar outcomes to what was observed from PHA (green) and protein (red). DNA staining gave a less pronounced definition of individual bacterial cell boundaries compared to protein staining. Notwithstanding, the DNA staining could still be used to observe and estimate the relative fractions and distribution of the biomass with respect to the total observed biomass that was participating in accumulating PHA.

FISH was applied to indicate distribution of overall microbial activity with respect to PHA accumulation activity. Distribution of RNA (purple in Figure 3a,e,j) suggested that the active fraction of individual flocs was already significant even by the start of the accumulation process. By 48 h of direct accumulation, nonactive biomass fraction could be discerned (blue in Figure 3a,e,j). These distributions for active and nonactive floc fractions were without an identifiable pattern or coupling specifically to PHA storage activity. In other words, no obvious spatial pattern of correspondence was observed between activity and PHA storage. Thus, an impression was that even if all PHA-accumulating microorganisms exhibited 16S rRNA activity, this activity was not exclusive. Other fractions of biomass exhibited 16S rRNA activity that was not coincident with PHA storage (purple in Figure 3a,e,j).

Microbial activity away from the distributed floc zones of PHA storage, and an estimated average yield of PHA production on acetate (0.26 ± 0.03 gCOD_PHA_/gCOD_Acetate_ at 48 h), are indicative of the presence of flanking microbial activity. A maximum yield due to PHA storage and no microbial growth is expected to be 0.75 gCOD_PHA_/gCOD_Acetate_.^40^ The biomass PHA content level became nevertheless stable between 27 and 48 h but substrate utilization efficiency for PHA production was low. Stable average biomass PHA content with ongoing microbial activity can be indicative of PHA storage concurrent with active microbial growth.^41^ However, since active biomass growth was not measurably significant, the latter part of the accumulation process was inefficient, due to maintained substrate demand, even if PHA content remained stable over a prolonged time.

### 3.3 Application of Selective Staining in an Activated Sludge: Morphology of Microorganisms

**Figure 4:**
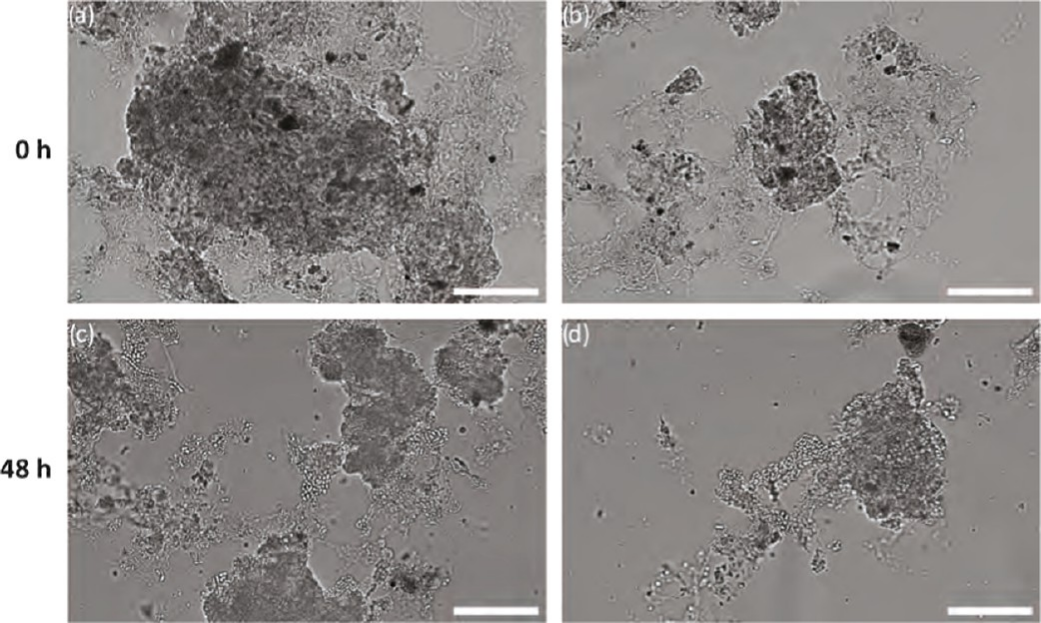
Bright-field images of typically observed floc structures after 0 h (a and b) and 48 h (c and d) direct accumulation. Scale bars represent 50 μm.

Well-functioning municipal activated sludge is dominated by biomass aggregates or floc structures. Different morphological structures in the flocs were observed using bright-field microscopy and CLSM during the direct accumulation experiments (Figures 2–4). Similar patterns of morphological diversity were observed in the replicate batches of waste activated sludge that were used. These structures included round amorphous compact microcolonies, irregular open structures abundant with filamentous microorganisms, low abundance of fingered zoogloeas, free-living filamentous and, also, planktonic cells (Figure 4). The majority of the flocs were found to be larger than 100 μm and with a thickness more than 100 μm. Larger flocs were seen to be bridged by filamentous bacteria, while the smaller flocs were not. The observed floc and cell morphologies were typical for municipal activated sludge.^42^ The morphological characteristics did not change during direct accumulation (Figure 4). Even though no active growth was observed when the VSS was measured, by 48 h, minor signs of growth were observed due to increased abundance of planktonic cells and cells that were loosely attached to the floc structures. Those newly appearing cells were with similar morphology, and most of them were also accumulating PHA. These observations were indicative of a balance of active growth for both PHA storing and nonstoring microorganisms later during the process.

Protein and DNA stained images provided better visualization of detail for distinguishing cell morphology than was possible from the bright-field images (Figures 2 and 3). By 27 h into the accumulation process, when PHA content reached a plateau level of 0.48 ± 0.02 gPHA/gVSS, two floc fractions became clearly delineated. PHA-accumulating microorganisms were bacterial cells, where the stained PHA granule structures overlapped with protein or DNA-stained structures. Microbial cells without stained PHA granule structures after 27 h could be interpreted as the non-PHA-accumulating biomass fraction.

**Figure 5:**
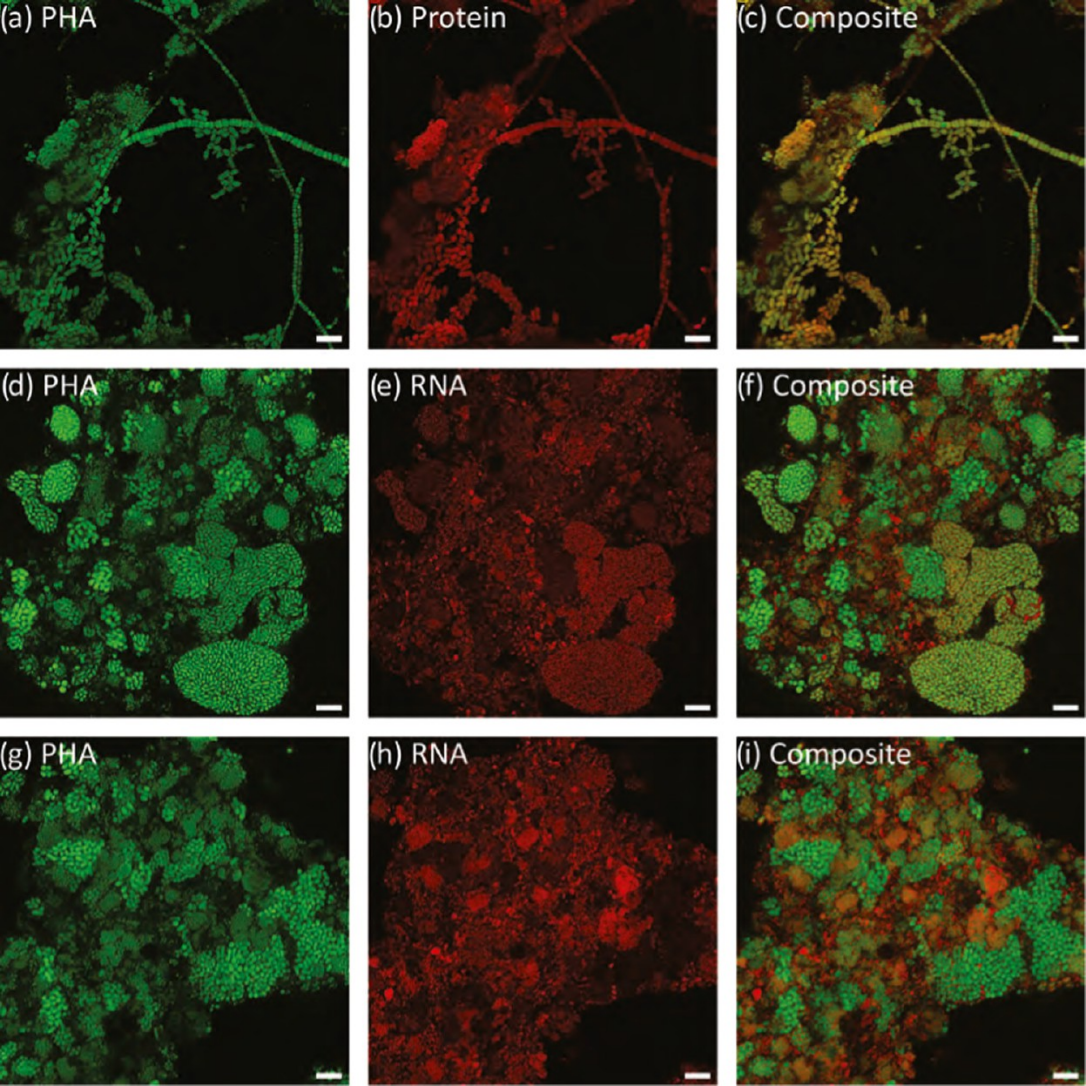
Morphology of PHA-accumulating microorganisms after 27 h direct accumulation with PHA biomass content at the saturation level of about 0.48 ± 0.02 gPHA/gVSS. Scale bars represent 10 μm.

Interestingly, the level of discrimination for cellular details evolved and improved with PHA storage (Figures 2 and 3). Specifically, individual cells became more readily discernible, and segmentation between cells in clusters became more clearly visible. Different levels of heterogeneity were observed between individual flocs and individual microbial cells. Clusters of populations of PHA-accumulating microorganisms were observed to be distributed heterogeneously among different flocs. Figure 5 shows illustrative examples of thin and thick filaments, cocci, rod-shaped and short-rod-shaped microorganisms that were observed to be accumulating PHA. Filamentous morphotypes that have been reported typically in activated sludge could not be easily classified especially before any PHA accumulation. Different types of thick filaments that are typical to municipal activated sludge were found to accumulate PHA. Those filaments were mainly smoothly curved within the floc structures, or free-living but bridging the flocs. Square and rectangular individual cells were the dominant shape in these cases. Barrel-shaped filaments were found less frequently. For all thick filaments, after 48 h direct accumulation, the cross-walls between cells became clearly visible.

Classification, e.g., by using specific probes providing morphotype identification, could be applied for added benefit in future work. Such probes would allow for further characterization and especially for following changes of the morphology before and after the PHA accumulation. Non-filamentous PHA-accumulating microorganisms sharing similar morphology were observed as distinct dense microcolonies. These islands of compact microcolonies represented the dominant observed fraction of the PHA accumulators distributed in the biomass. The clustered morphotype suggested that dominant PHA-accumulating species or families might exist in the municipal activated sludge. However, assessment of dominant PHA-accumulating families or species needs to be further evaluated by means of molecular phylogenetic measurements. Specific FISH probes could be applied, but this step was beyond the scope of the present investigation focused on the counterstaining method development for PHA distribution and degree of enrichment visualization.

The degree of heterogeneity in distribution and the pockets of more intense activity for PHA storage were unexpected. A significant distinct fraction of the biomass was observed that was not accumulating PHA. Measurements of average dried solids biomass PHA content will not reveal this heterogeneity because the analyzed sample size is too large. Since two significant distinct biomass fractions were observed, the average biomass PHA content in the PHA-accumulating fraction is expected to be significantly higher. If the fractions are separable, then a biomass with much higher PHA content could be harvested as part of the downstream processing for polymer recovery. The quantification of the PHA-accumulating fraction in PHA-rich biomass was further evaluated and reported by Pei et al.^43^

The volatile suspended solids, together with PHA content measured by TGA measurements, indicated that there was no significant active growth of the microorganisms during the 48 h of PHA accumulation process. Image resolution was sufficient to estimate the size of the PHA-accumulating bacteria. Thin filamentous and coccus-like microorganisms with a diameter smaller than 0.5 μm did not evolve with obvious size changes due to stored polymer. However, the sizes of other PHA-accumulating microorganisms did increase, especially in length and width of rod-shaped microorganisms, and width of the thick filaments. The size of the rod-shaped microorganisms was between 0.5 and 1.0 μm or smaller before the accumulation. The size of those initially rod-shaped microorganisms increased to about 2–3 μm with granules. PHA granules caused distortions to the morphotypes that made native rod or coccus organisms, with dimensions smaller than 2 μm, and typically around 0.5 μm, become morphologically indistinguishable. The length of filaments could become longer and more than 100 μm. The width of the thick filaments doubled from about 0.5–1 μm for the square and rectangular types. The barrel-shaped filaments had a width of around 2 μm after 48 h.

However, change in cell size and morphology was not evident for all types of cells in the PHA-accumulating fraction of the biomass. Thus, two distinct groups of PHA-accumulating microorganisms were observed for this specific activated sludge. In one group, cell sizes increased significantly due to PHA storage, while in the other, they remained the same. The first group exhibited adaptive stretchability. This expandability suggested reduced cell wall stiffness. The other groups were more rigid and thus maintained an apparently higher degree of cell wall stiffness. Rigid cells are reported to express a lower capacity to store PHA.^44,45^ The biomass PHA content in the PHA storing fraction may, therefore, be bimodal, with rigid cell types having lower accumulation potential than those organisms exhibiting significant expandability. Heterogeneity of biomass rigidity also can influence considerations important for polymer recovery. During the post-accumulation downstream processing, stretched (stressed) cells may be more susceptible to lysis due to disturbances (shear forces or chemical pretreatments). Polymer may be easier to recover from the stressed distended biomass fraction, but also easier to lose in the process. The opposite may be expected when recovering polymer stored from rigid cell structures. Rigid cells may not lyse so readily, and purification of the polymer through washing steps may be less effective. PHA recovery and purification methods after a direct accumulation process will need to address a challenge to reach outcomes for polymer yield and quality that are optimal (or a compromise) for all cell types that are present in the biomass.

**Figure 6:**
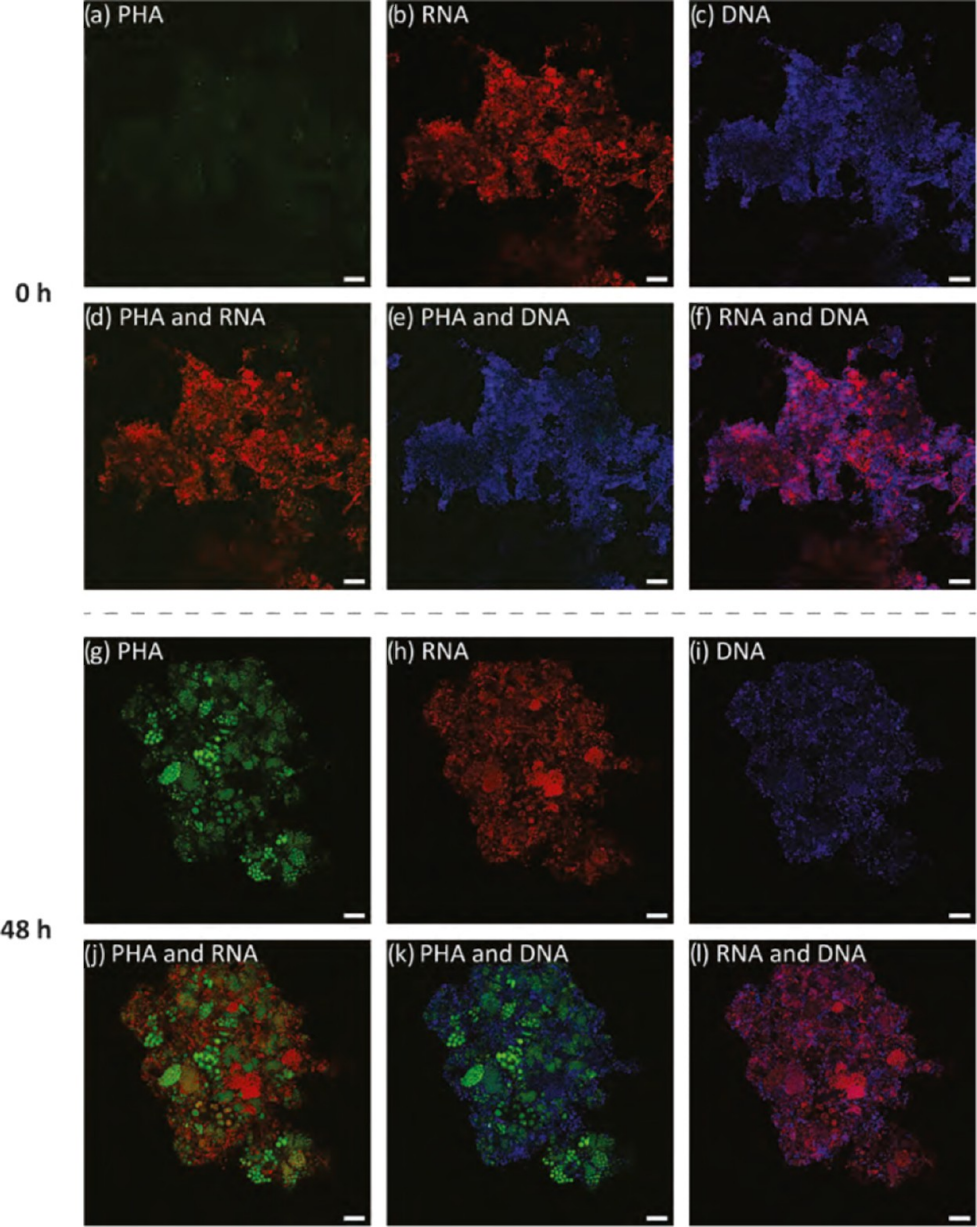
Distributions of PHA (green), RNA (red), DNA (blue), and the composites initially (a–f) and after 48 h (g–l) direct accumulation experiments. Scale bars represent 10 μm.

The distribution of RNA and DNA was also influenced by PHA granule storage (Figure 6). Before direct accumulation, RNA and DNA were more evenly distributed in the cell (Figure 6a–f). However, after 48 h accumulation, DNA and RNA were observed to be displaced and crowded out within the cytoplasm due to the granules (Figure 6j,k,l). Before PHA accumulation, RNA was distributed uniformly over the whole cell. After PHA accumulation, RNA was observed to distribute like a ring surrounding the PHA granules. It was found that the DNA of the cells was pushed even further to a different focus plane compared to the RNA. Thus, where there was PHA accumulated (Figure 6g,h,j), there was no DNA signal (Figure 6g,i,k) in the same area. PHA storing microorganisms are known to be able to store PHA concurrent to cell division.^46^ It is interesting to consider further how or if this change in the distribution of vital cellular machinery influences function.

**Figure 7:**
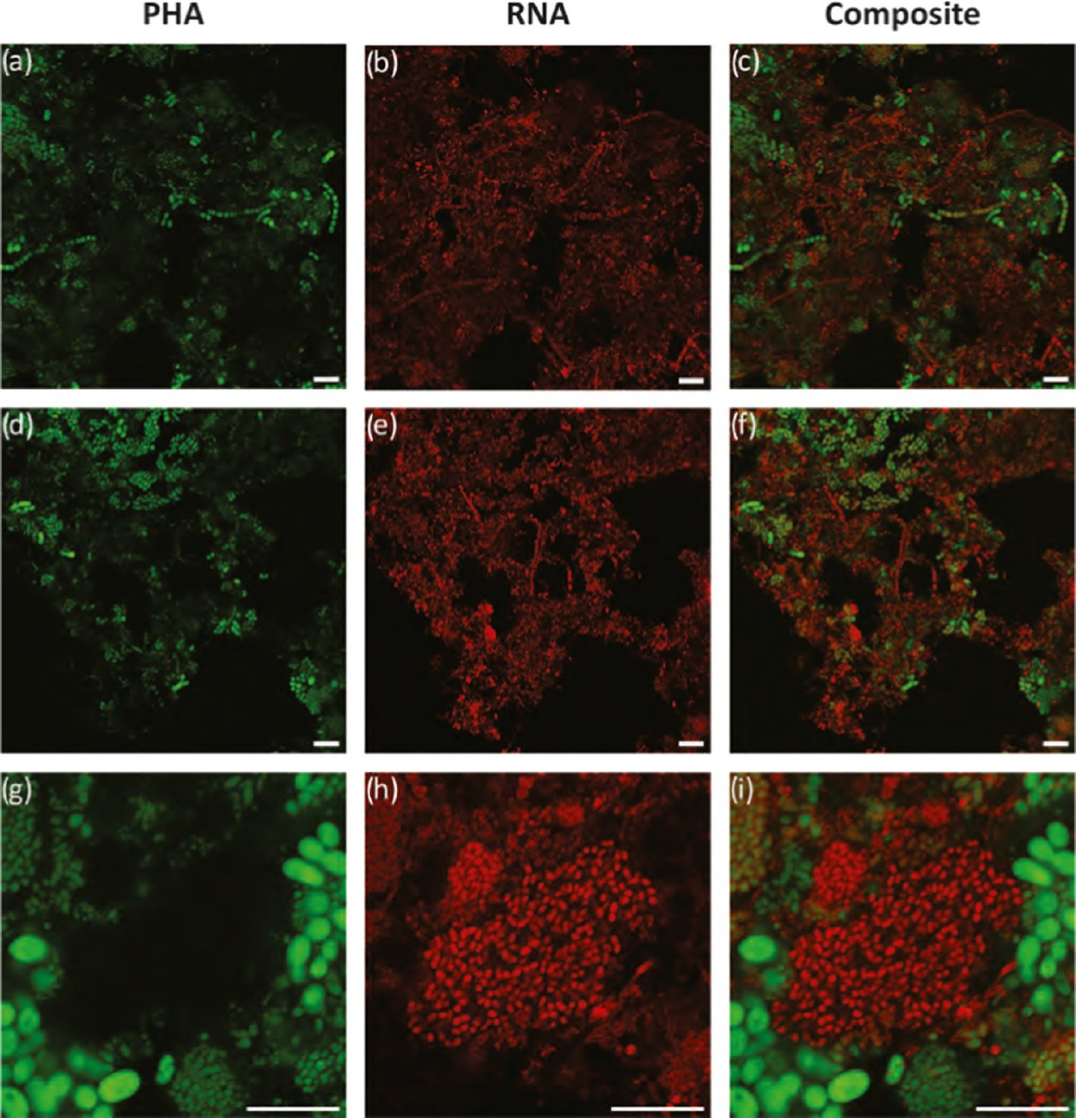
Morphology of PHA-accumulating (green overlay with red) and non-PHA-accumulating microorganisms (only red) from direct accumulation experiments. Scale bars represent 10 μm.

Non-PHA-accumulating microorganisms were thin filaments and rod-shaped cells (Figure 7). The rod-shaped microorganisms without PHA granules were not considered to be unique or distinct in morphology from the rod-shaped PHA-accumulating microorganisms. It was also found that some of the non-PHA-accumulating microorganisms also formed clusters and could be characterized to be forming less compact flocs. Organic substrate supply with limiting nutrients can promote PHA storage as well as excess EPS production.^47^ With an excess in supply of organic carbon, there are two distinct possible responses for an activated sludge, which are PHA accumulation or EPS production. The EPS formation would lower the yield of PHAs on the substrate. In continued work, it would be of value to further explore the ecophysiology roles of EPS versus PHA, and any competitive strategy for the non-PHA-accumulating microorganisms during direct accumulation. If that activity can be mitigated, then volumetric productivity can be increased significantly. Alternatively, the understanding can be directed to augmenting selection pressure during the wastewater treatment. Increased selection pressure would reduce the fraction and activity of the nonstoring biomass in direct accumulation.

### 3.4 Application of Selective Staining in an Activated Sludge: Distribution of PHA Granules within Individual Cells

**Figure 8:**
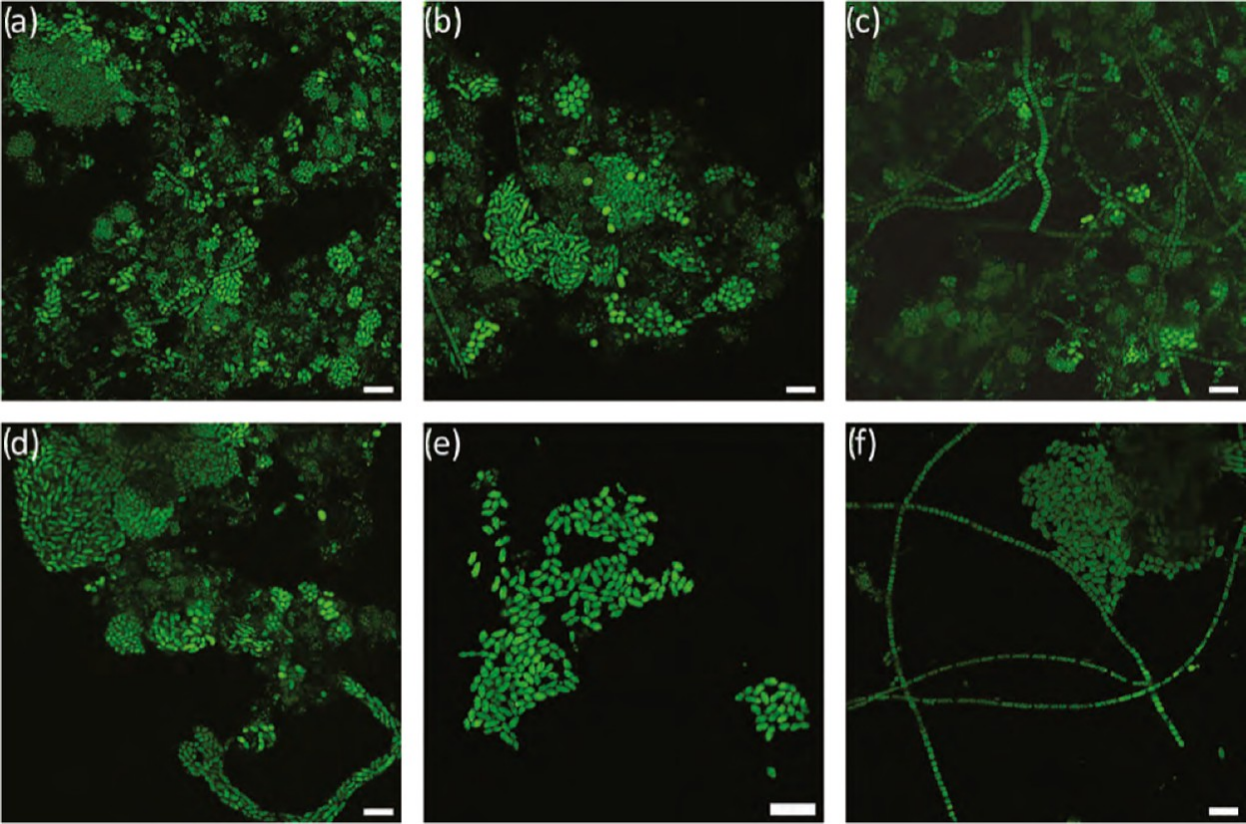
Morphology differences for PHA granules stored in the waste activated sludge from replicate direct accumulation experiments. Scale bars represent 10 μm.

Selective staining in combination with CSLM enabled resolution of details to discriminate between floc morphologies. In some cases, resolution was to the level of detail of PHA granule morphology. Section 3.3 reported how similar morphotypes of PHA-accumulating microorganisms were found to be clustered. The fluorescent emission of the stained PHA granule covered contiguous floc areas. An impression from replicate experiments was that similar morphotypes progressed similarly in the buildup of stored PHA content. It was also observed that different clusters of morphotypes accumulated distinctly different estimated sizes and numbers of the PHA granules per cell. Seven different types of PHA granule morphologies were identified, as illustrated in Figure 8. In some cases, individual PHA granules could be distinguished quite clearly. This level of detail that typically requires transmission electron microscopy suggests a high degree of selectivity for PHA staining using BODIPY. For cocci and rod-shaped bacteria, smaller cells were seen to store smaller individual PHA granules, as also found for pure culture rod-shaped bacteria like *Ralstonia eutropha* and *Cupriavidus necator*.^45^ Observations for PHA granules in filaments were similar to those reported by Dionisi et al.^48^ The number of granules per cell varied and was estimated to be from 3 to more than 10, as is typical for pure cultures.^45^ Ultimately, size and number of PHA granules can become limited due to cell size.^44^

### 3.5 Implication of Selective Staining Methods

This case study showed the application of selective staining in a PHA direct accumulation process. Insights from details were revealed from the visualization of PHA development and its distribution. The applied staining methods provided a tool for morphological analysis of PHA-accumulating microorganisms for a diverse biomass and in a complex floc matrix. The quantitative aspect of the staining method was discussed, and the staining method was applied to PHA-rich biomass procured from different sources of activated sludge.^43,49^ Similar methods could also be applied for comparing the PHA production using enrichment accumulation to monitor and better understand process and production methods in general. Further, such staining may be applied as a diagnostic tool to confirm or understand the fate of PHA granules and biomass fractions during downstream processing.^50^

PHA is not only a product of interest for resource recovery from waste. It is also a central metabolic intermediate as a storage polymer in wastewater treatment using activated sludge or aerobic granular sludge. The roles and dynamics of PHA-accumulating microorganisms such as polyphosphate-accumulating organisms (PAOs) and glycogen-accumulating organisms (GAOs) strongly influence process performance in biological phosphorous removal. However, PAOs and GAOs are not readily isolated, hampering developments in understanding of population dynamics and physiology. The presented staining method could be extended and used to visually reveal the dynamics of PHA storage for wastewater treatment in general. The experience from undertakings in the present work suggests that selective staining with systematic visualization by CSLM can provide perspectives toward, for example, activated sludge modeling and/or insights into process challenges and improvements from routine diagnostic assessments. It is a tool to help with monitoring and advancements for wastewater treatment plants.^51^

## Supporting Information Available

PHA staining with Nile blue A and Nile red (Figure S1) and evaluation of protein and DNA staining as the counterstaining (Figure S2) (PDF)
