# Supplementary material for: Visualization of Polyhydroxyalkanoate Accumulated in Waste Activated Sludge
Source: Environ Sci Technol. 2023 Jul 20;57(30):11108–21. doi: 10.1021/acs.est.3c02381 (PMC10399201; doi:10.1021/acs.est.3c02381)
Supplement: Supplementary file 1 — es3c02381_si_001.pdf [file es3c02381_si_001.pdf]

Visualisation of Polyhydroxyalkanoate Accumulated in Waste Activated Sludge

Ruizhe Pei [1,2,*], Gerard Vicente-Venegas [2], Agnieszka Tomaszewska-Porada [2], Mark C.M. Van Loosdrecht [1], Robbert Kleerebezem [1], Alan Werker [2]

[1] Department of Biotechnology, Delft University of Technology, Van der Maasweg 9, 2629 HZ Delft, the Netherlands

[2] Wetsus, European Centre of Excellence for Sustainable Water Technology, Oostergoweg 9, 8911 MA, Leeuwarden, the Netherlands

[*] Corresponding author; r.pei@tudelft.nl

This supporting information contains 2 pages and 2 figures.

Supporting Information

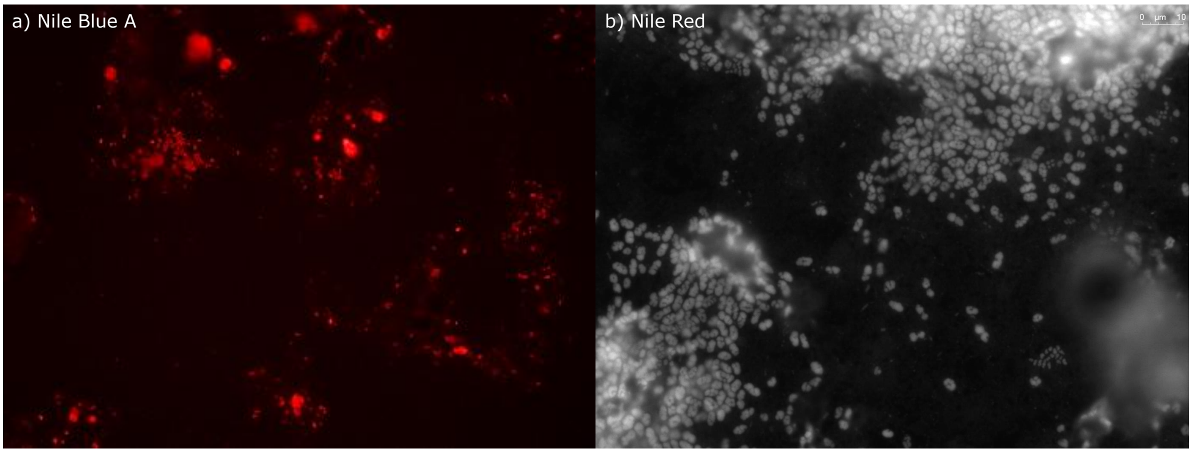

Figure S1: Visualisation of PHA accumulation in activated sludge after 24 hours by staining with Nile blue A (a) and Nile red (b).

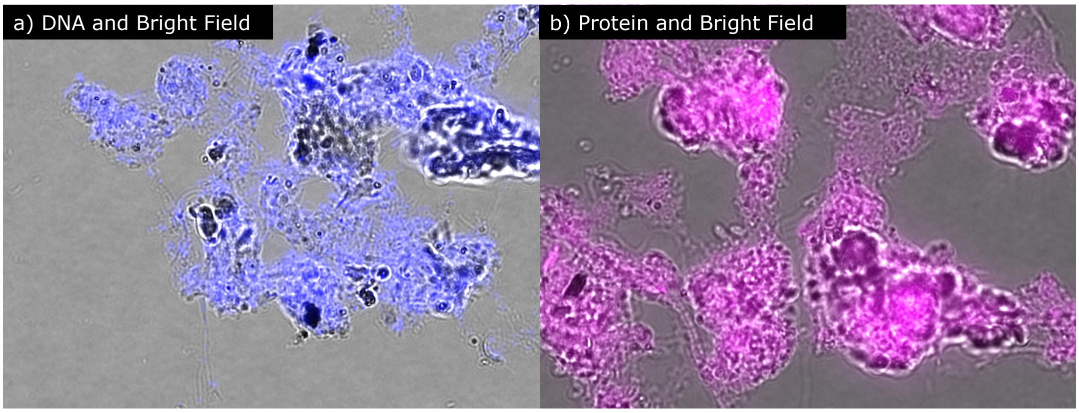

Figure S2: Bright field evaluation of the effectiveness of DNA (left in blue) and protein (right in pink) staining of activated sludge samples comparing with Bright field.
